# Arbovirus surveillance in febrile patients attending selected health facilities in Rwanda

**DOI:** 10.1080/20008686.2023.2289872

**Published:** 2023-12-07

**Authors:** Vincent Rusanganwa, Olivia Wesula Lwande, Brenda Bainda, Patrick I. Chiyo, Eric Seruyange, Göran Bucht, Magnus Evander

**Affiliations:** a College of Medicine and Health Sciences, University of Rwanda, Kigali, Rwanda; b Department of Clinical Microbiology, Umeå University, Umeå, Sweden; c Teaching Coordination and Quality Assurance Department, Ministry of Health, Kigali, Rwanda; d Department of Biology, Duke University, Durham, North Carolina, USA; e Internal Medicine Department, Rwanda Military Hospital, Kigali, Rwanda

**Keywords:** Tick-borne viruses, mosquito-borne viruses, molecular detection, arbovirus, Rwanda

## Abstract

Arthropod-borne (arbo) viruses cause emerging diseases that affect the livelihoods of people around the world. They are linked to disease outbreaks resulting in high morbidity, mortality, and economic loss. In sub-Saharan Africa, numerous arbovirus outbreaks have been documented, but the circulation and magnitude of illness caused by these viruses during inter-epidemic periods remains unknown in many regions. In Rwanda, there is limited knowledge on the presence and distribution of arboviruses. This study aimed at determining the occurrence and distribution of selected arboviruses, i.e., chikungunya virus (CHIKV), o’nyong-nyong virus (ONNV), dengue virus (DENV), West Nile virus (WNV), Zika virus (ZIKV), Rift Valley fever virus (RVFV) and Crimean-Congo haemorrhagic fever virus (CCHFV), among febrile patients visiting health centres in Rwanda. A total of 2294 dry blood spots (DBS) were collected on filter papers during August 2019 – December 2020. Reverse-transcription polymerase chain reaction (RT-PCR) was performed on samples in pools of ten, using both quantitative (DENV, ZIKV, RVFV) and conventional PCR (CHIKV, ONNV, WNV, CCHFV) with virus specific primers, followed by sequencing. Demographic data and clinical manifestations of illness were analysed. ONNV infection was detected in 12 of 230 pools (5.2%) and ZIKV in three pools (1.3%). The other arboviruses were not detected. All ONNV cases were found in the Rwaniro health centre, while ZIKV infection was found among patients visiting the Kirinda and Zaza health centres. There was temporal variability in ONNV infections with most cases being recorded during the long dry season, while ZIKV infection occurred during both dry and wet seasons. Patients with ONNV were older and more were females. In conclusion, ONNV and ZIKV infection were detected in acute patients and can explain some of the feverish diseases in Rwanda.

## Introduction

Arboviral outbreaks of chikungunya virus (CHIKV), o’nyong-nyong virus (ONNV), dengue virus (DENV), West Nile virus (WNV), Zika virus (ZIKV), Rift Valley fever virus (RVFV), and Crimean-Congo haemorrhagic fever virus (CCHFV) are increasingly becoming a common occurrence in Africa [1–3]. Improved arbovirus surveillance is critical especially in Africa where most epidemics have been reported [4]. The sporadic nature of these outbreaks poses a challenge to effective surveillance and early diagnosis since most of them have already resulted in high morbidity and mortality [5–7]. Moreover, they cause strain on the economy of affected countries, because resources allocated for development projects are instead used to tackle the burden of the diseases [8]. Considering most of the outbreaks are sporadic and occur in poor resource settings, there is a need to establish well-structured early warning and response systems that can enable prediction and detection of the viruses before they spread to large geographical areas. Through regular surveillance, arboviruses can be monitored to trace their circulation and population dynamics, which is key in informing public health decisions for risk planning, rapid response, and effective control.

For most arboviruses, there are no vaccines or antiviral therapeutics [9]. In addition, several of the arboviruses present with similar clinical manifestations, which makes correct clinical diagnosis difficult. In addition, diagnostic assays that detect antibodies against these viruses, tend to cross-react, which makes it difficult to distinguish between virus infections. The lack of appropriate sensitive diagnostic kits that can target the specific viruses may result in under- and misdiagnosis, leading to poor patient management.

Since 2010, there has been an ongoing viral haemorrhagic fever virus (VHF) surveillance system entailing sentinel surveillance in Rwanda, South Sudan, and Uganda [10]. Through the surveillance, there have been multiple confirmed cases and outbreaks of the tick-borne arbovirus CCHFV [11]. In addition, between 2017 and 2020, the East African Community (EAC) comprising of six countries (Burundi, Rwanda, Tanzania, Kenya, Uganda, and South Sudan), established nine Biosafety Level (BSL) 3/4 mobile network laboratories which have helped reduce the turn-around-time for responding to outbreaks [12]. Diagnostics platforms were established to screen for several pathogens including CCHFV, CHIKV, DENV, RVFV, WNV, and Yellow fever virus [12]. However, despite the commissioning of the program, the prevalence of arboviruses in Rwanda remains unknown. Moreover, there is not sufficient capacity to detect and tackle arboviruses at the grass root level. In a recent study conducted in Rwanda on clinical referral laboratories, 73% (*n* = 492) of physicians from the four main referral hospitals expressed their concerns of lack of the capacity of doing viral tests in their clinical practice and advocate for increasing of this capacity in referral laboratories to detect common viruses circulating in the country [13]. The current study aimed at performing surveillance of seven arboviruses, ONNV, ZIKV, CCHFV, CHIKV, DENV, RVFV, and WNV among febrile patients attending selected health centres in Rwanda. The study will contribute to knowledge of arbovirus incidence in Rwanda and guide diagnostic preparedness.

## Materials and Methods

### Ethical considerations

The research proposal was approved by the Rwanda National Ethics Committee (reference numbers 0059/RNEC/2017, 111/RNEC/2018, and 096/RNEC/2020) and the Rwandan Ministry of Health. The study involved patients aged 18 years and above. Participation was voluntary and informed consent obtained.

### Study setting and sampling

The health-care system in Rwanda is a bottom-up referral system and a health centre is a primary health-care facility, which is the first contact facility for patients. It refers patients to district hospitals, which can then forward them to referral hospitals, once not managed at this level. A health centre in Rwanda serves an average of 25,771 people in 2022 and is managed by professional nurse. The laboratory at health centre is headed by laboratory technician. In the health centre, patients receive curative, preventive, and promotive health services provided by nurses and midwives, and unmanaged cases are referred to a district hospital.

**Figure 1.**
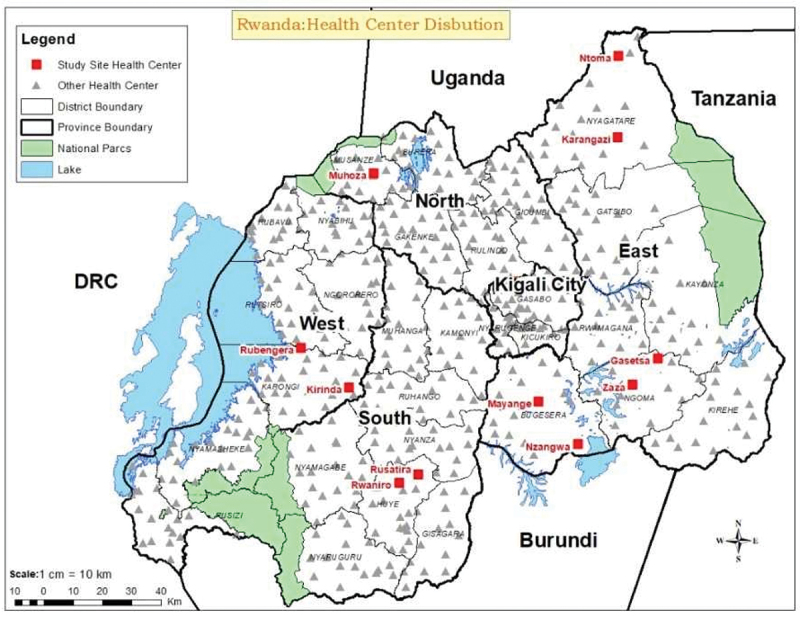
Map of Rwanda indicating the selected health centres sampled in the study (red boxes).

We targeted primary health-care facilities as mainly first contact of health visit to increase the possibility of having the febrile patients. Administrative districts were purposively selected from four provinces. Three administrative districts in the Eastern Province (Bugesera, Ngoma, and Nyagatare districts) and one district per province in the remaining three provinces (Huye, Karongi, and Musanze in Southern, Western, and Northern Provinces, respectively) were selected based on logistics such as easier accessibility and transportation. Based on high prevalence of malaria cases, a mosquito borne disease, using Rwanda health management information system data, health centres with a high malaria prevalence in a district were selected with the hypothesis that this malaria prevalence would coincide with a high frequency of mosquitoes and mosquito-borne pathogenic viruses. In each district, we selected two health centres, except for Musanze District, where only one health centre was selected, as the prevalence of malaria is not high in this region. The tick-borne CCHFV was also included in the analysis, due to prevalence in the East Africa region. The distribution of selected health centres is indicated in Figure 1.

### Study design and data collection

The cross-sectional study was performed during different seasons to account for weather variations that could affect mosquito prevalence and thus arbovirus infections. Data collection was carried out in the period from August 2019 to December 2020. Patients presenting with fever (temperature equal or more than 37.5°C measured in axillary) were included in the study, while patients confirmed with malaria were excluded. After the consent of each participant, patients’ disease history (duration of illness, fever duration, presence of joint pain and peripheral neuropathy), age and gender were collected using a structured questionnaire. Then, vein blood sample was collected in a dry tube from patients who accepted to participate in the study. Nobuto filter strips (ColeParmer, Vernon Hills, Illinois, USA) were dipped into the tube, and the strips were dried for 24 h at room temperature as previously described by Näslund J et al. [14]. Thereafter, the dry blood spot samples (DBS) on the filter strips, were placed in a zipped plastic bag with desiccant and kept at −20°C. They were then transported to Kigali, Rwanda to be kept at −80°C, from where they were transported in a cold-chain to Umeå University, Sweden to be kept at −80°C.

### Sample processing

The DBS were processed using sterile surgical blades (Swann-Morton®, Sheffield, England). To limit contamination, each filter paper was cut using a new sterile surgical blade and eluted separately in 2 ml micro tubes (SARSTEDT) containing 1 mL of phosphate-buffered saline – Tween-20. The tubes were incubated overnight on a shaking rack at 4°C. After the incubation, the eluted samples were stored at −80°C for later use. To reduce processing time and cost, 230 pools containing 10 samples per pool, were generated, by adding 100 µl/sample into a micro tube.

### RNA extraction and cDNA synthesis

Extraction of viral RNA was performed using QIAmp® Viral RNA Mini Kit (QIAGEN, Hilden, Germany). This procedure was performed according to the manufacturer’s protocol (Spin Protocol), where 140 μL of each virus homogenate was used as a sample volume to generate 60 μL of viral RNA. The extracted RNA was converted to cDNA using the Revert Aid RT kit (Thermo-Fisher Scientific Vilnius, Lithuania), according to manufacturer’s instructions.

### Amplification of selected arboviruses by conventional reverse transcriptase polymerase chain reaction (RT-PCR) and by quantitative reverse transcriptase polymerase (qRT-PCR)

CHIKV, ONNV, WNV, and CCHFV were amplified using conventional RT-PCR. The PCR master mix was prepared using the Phusion Green Hot Start II High-Fidelity PCR Master Mix (Thermo Fisher Scientific, Vilnius, Lithuania) with respective virus primers and their target genes (Table 1). For each reaction, 2 µL of template was used together with 10 µL of 2× Phusion mix, 1 µL of both forward and reverse primers (10 pmol), 0.6 µL of DMSO and 4.4 µL of nuclease free water, up to a total reaction volume of 20 µL. As negative and positive controls, 2 µl of nuclease free water and virus-specific RNA were used, respectively. Conditions for reactions were 98°C for 30 s for initial denaturation followed by amplification using 35 cycles of denaturation at 98°C for 7 s, annealing at 60°C for 15 s, and extension at 72°C for 20 s. The final extension was performed at 72°C for 7 min.

**Table 1.** Description of CCHFV, CHIKV, ONNV, DENV, RVFV, WNV, and ZIKV primers.

Virus	Primer name	Sequence 5’ to 3’	Target gene	Source
CCHFV	CCHFV-F2	CTCAAAGAAACACGTGC CGCTTAC	Nucleocapsid gene	This study
	CCHFV-R155	AGTTTGTGAAGGTGTCC ACAAGTCC	Nucleocapsid gene	This study
CHIKV	CHIKV 2F without tail	GGCAACGTAAAGATCACAGTCA	Partial structural genes E2/E1	[9]
	ONNV/CHIKV 2 R	TTGCAGTTATTWATSACTTTGTC	Partial structural genes E2/E1	[9]
ONNV	ONNV 2F	GGGGCGGGGCGGGGGGGGCGGAAACGTTAAGATCACAGTTG	Envelope protein E2	[9]
	ONNV/CHIKV 2 R	TTGCAGTTATTWATSACTTTGTC	Partial structural genes E2/E1	[9]
DENV	Den238 forward	ATTAGAGAGCAGATCTCTG	Serotypes 1, 2 and 3 polyprotein (POLY) gene	[15]
	Den239 probe	6FAM-TCAATATGCTGAAACGCG-BHQ1	Serotypes 1, 2 and 3 polyprotein (POLY) gene	[15]
	Den240 reverse	TGACACGCGGTTTC	Serotypes 1, 2, & 3 polyprotein (POLY) gene	[15]
RVFV	qPCR RVF forward	TGAAAATTCCTGAGACACATGG	RVFV segment L polymerase gene	[16]
	qPCR RVF reverse	ACTTCCTTGCATCATCTGATG	Segment L polymerase gene	[16]
	qPCR RVF probe	6FAM-CAATGTAAGGGGCCTGTGTGGACTTGTG-BHQ1	Segment L polymerase gene	[16]
WNV	WNVV1-F1	TTGGAATGAGCAACAGAGACTTCTTG	Envelope (E) gene	This study
	WNVV1-R149	GCCTCCATATTCATCATCTTCACATC	Envelope (E) gene	This study
	WNVV2-F572	AGCCACGGTCAGGAATAGACAC	Envelope (E) gene	This study
	WNVV2-R683	GCACTGCTCCATGGCAGGTTC	Envelope (E) gene	This study
ZIKV	qPCR ZIKV forward	AAGTTTGCATGCTCCAAGAAAAT	Envelope (E) gene	[17]
	qPCR ZIKV reverse	CAGCATTATCCGGTACTCCAGAT	Envelope (E) gene	[17]
	ZIKV probe	ACCGGGAAGAGCATCCAGCCAGA	Envelope (E) gene	[17]

For amplification of DENV, ZIKV, and RVFV we used quantitative PCR (qRT-PCR). qRT-PCR for DENV, ZIKV, and RVFV was performed using qPCRBIO SyGreen 1-Step Go Hi-ROX kit (PCR Biosystems Ltd, London, UK). Briefly, for each individual reaction, 6 µL of nuclease free water, 10 µL of 2× qPCRBIO Probe 1-Step Go mix, 0.8 µL each of forward and reverse primers (10 pmol), 0.4 µL of 10pmol probe (Table 1) and 1 µL of 20× RTase Go mix were added to produce the master mix. Then, 18 µL of the master mix was added into duplicate wells in a 96 well plate. In addition, 2 µl of negative (nuclease free water) and positive controls (viral RNA) were added to separate wells. The plate was centrifuged at 1700 rpm at 4°C for 2 min. The RT-qPCR was performed using the StepOne software protocol. The cycling conditions were one cycle at 45°C for 10 min and 95°C for 5 s, followed by 40 cycles of 95°C for 5 s and 60°C for 30 s.

### Detection and visualization of positive arbovirus samples

The RT-PCR and qRT-PCR products were visualized by gel electrophoresis using 3% agarose in 1× Tris-Acetate-EDTA with GelRed (Biotium Inc. Hayward, CA, US). We used the negative control to check for contamination as indicated by the absence of a band. The positive control was used to confirm positive samples by matching band sizes with the control, following gel electrophoresis.

### Sequencing of PCR-positive products

ONNV positive PCR products were purified with ExoSAP-IT kit (Thermo Fisher Scientific, Vilnius, Lithuania) and sent to Eurofins Genomics (Germany) for Sanger sequencing. The sequences were edited using Geneious software (Geneious Prime® 2023.0.2 Build 9 January 2023 11:52). The resulting sequences were then aligned to previously identified virus strains in GenBank database using the Basic Local Alignment Search Tool (BLAST). Trimmed ONNV Sequences obtained during this study are provided as supplementary material.

### Data analyses

Summary descriptive statistics of age, body temperature, fever duration, and illness duration of all patients were calculated for the study population and by health centre. The number of patients having neuropathy and joint pain overall and by health centre was also calculated. To determine the variation in prevalence of pathogens causing febrile illness by health centre we used the epiR package in the R statistical computing software [18,19]. The association between arbovirus prevalence across locations, gender, age, and occupation are presented, but not statistically tested because of potential confounding by nonpositive individuals in pools. Similarly, we also present data on variation in symptoms such as fever, arthralgia, and neuropathy across patients in negative and positive pools without statistical testing.

## Results

### Demography

2284 of a total of 2294 patient samples, had complete metadata. The mean ± SD of age for patients sampled was 40.5 ± 16.3 years (median = 39.2, range: 18–101, *n* = 2284). There was variation in age among patients sampled from different health centres (Table 2). The highest mean ± SD age was 48.4 ± 17 (range: 18–94, *N* = 385) from the Zaza health centre, and the lowest mean age was 29.2 ± 9.2 years (range: 18–54, *N* = 58) from the Muhoza health centre. Most patients (62%, *n* = 1424) were females. There was variation in sex distribution among health centres (Table 2). The Rwaniro health centre had three times more females than males. The Gasetsa, Kirinda, and Nzangwa health centres had twice more females than males, whereas Mayange, Karangazi, Rubengera, and Rusatira had a similar frequency (Table 2). Most patients were farmers (86%, *n* = 1964), followed by office workers (4.6%, *n* = 105), and vocational workers (3.2%, *n* = 72). The ‘other’ occupation constituted 6.3% (*n* = 143).

**Table 2.** Demographic characteristics of febrile patients from selected health centres in Rwanda.

Health centre	N	Mean age ± SD	Sex (Male: Female)	Farmer (%)	Office worker (%)	Other (%)	Vocational Worker (%)
Gesetsa	302	38.6 ± 14.8	1: 2.4	96.7	0	3.3	0
Karangazi	184	40.5 ± 16.1	1: 1.1	38.6	23.9	20.1	17.4
Kirinda	197	40.7 ± 17.0	1: 2.1	87.8	2.0	7.1	3.1
Mayange	29	33.8 ± 8.5	1: 1.1	27.6	69.0	0	3.6
Muhoza	58	29.2 ± 9.2	1: 1.6	65.5	15.5	17.2	1.7
Ntoma	266	37.2 ± 15.3	1: 1.8	90.2	3.4	4.5	1.9
Nzangwa	287	37.9 ± 15.5	1: 2.1	97.9	0	0.4	1.7
Rubengera	193	33.9 ± 14.1	1: 1.1	70.5	2.1	25.4	2.1
Rusatira	147	40.1 ± 15.5	1: 1	91.2	6.1	2.7	0
Rwaniro	236	45.9 ± 16.6	1: 3.4	97.9	0	2.1	0
Zaza	385	48.4 ± 17.0	1: 1.2	93.5	1.6	0.3	4.7
Total	2284	40.5 ± 16.3	1: 1.7	86.0	4.6	6.7	3.2

### Detection and prevalence of arboviral infections

The PCR results revealed the presence of ONNV and ZIKV RNA among febrile patients. No CCHFV, CHIKV, DENV, RVFV, and WNV RNA was detected. ONNV infection was most common and was detected in 12 of the 230 pools examined, resulting in a pool positivity rate of 5.2% (95% CI: 5.2–8.9%). ZIKV was detected only in three pools (1.3%; 95% CI: 0.27–3.8%). The results were confirmed by sequencing the PCR products from the 12 ONNV positive samples. Of these, 8 resulted in clean editable sequences of around 70bp after trimming (Supplementary material). Three haplotypes were recovered (Table 3). All the three haplotypes had a 100% sequence similarity ONNV sequence AF079456 (SG6560 isolated from Uganda during the 1996/97 ONNV epidemic).

**Table 3.** Blast results for ONNV sequences obtained from the NCBI database.

Sequence ID	GenBank ID	Matching species ID	Percent Identity	E- Value	Query Coverage
Haplotype 1					
EGK294	AF079456	O’nyong-nyong virus strain SG650	100%	0.00	72.86
EGK292	AF079456	O’nyong-nyong virus strain SG650	100%	0.00	72.86
Haplotype 2					
EGK269	AF079456	O’nyong-nyong virus strain SG650	100%	0.00	72.82
EGK275	AF079456	O’nyong-nyong virus strain SG650	100%	0.00	72.82
EGK296	AF079456	O’nyong-nyong virus strain SG650	100%	0.00	72.82
Haplotype 3					
EGK271	AF079456	O’nyong-nyong virus strain SG650	100%	0.00	72.86
EGK273	AF079456	O’nyong-nyong virus strain SG650	100%	0.00	72.86
EGK410	AF079456	O’nyong-nyong virus strain SG650	100%	0.00	72.86

There was variation in positivity by health centre for both ONNV and ZIKV. Fifty percent (50%) of the pools obtained from patients attending the Rwaniro health centre were ONNV positive. ZIKV was detected in two health centres, Kirinda and Zaza, where the frequency of positive pools was 9.1% and 2.4%, respectively.

### Demographic factors and clinical symptoms in relation to infection with ONNV and ZIKV

Sample pools positive for ONNV were generally from older patients than pools from uninfected patients (Table 4). In terms of sex, all (100%) positive pools had at least a female whereas 60% of the pools had at least a male. Of the positive pools, 77.5% patients consisted of females, while in negative pools, 61.2% of individuals were female (Table 4). There were more farmers among positive pools for ONNV (99.17%, *n* = 119, *N* = 120) compared to office (0.83%, *n* = 1, *N* = 120) or vocational workers (0%, *n* = 0, *N* = 120). For ZIKV, there were no differences in age between individuals in positive compared to negative pools (Table 4). However, there was a significant association with sex with a greater proportion of males than females in positive pools (Table 4).

**Table 4.** Demographic factors and clinical symptoms in relation to o’nyong-nyong and Zika virus infection status (*N* = 230 pools).

Covariate	Total population	Positive pools	Negative pools
**O’nyong-nyong virus**			
Mean age (years) ± SD	40.5 ± 16.3	48.5 ± 18.2	40.1 ± 16.1
Sex: proportion of patients that are females	0.620	0.775	0.612
Mean temperature (^o^C) ± SD	38.1 ± 0.5	38.5 ± 0.7	38.0 ± 0.5
Mean fever duration (days) ± SD	2.5 ± 2.1	2.7 ± 1.9	2.4 ± 2.1
Mean illness duration (days) ± SD	3.6 ± 3.8	3.6 ± 2.3	3.6 ± 3.9
Joint pain: proportion of patients reporting symptom	0.218	0.742	0.189
Peripheral Neuropathy: proportion of patients reporting symptoms	0.045	0.500	0.020
**Zika virus**			
Mean age (years) ± 95% Confidence Interval	40.5 ± 16.3	44.2 ± 16.0	40.5 ± 16.3
Sex: proportion of patients that are females	0.623	0.364	0.630
Mean temperature (^o^C) ± SD	38.1 ± 0.5	37.9 ± 0.4	38.1 ± 0.5
Mean fever duration (days) ± SD	2.5 ± 2.1	2.0 ± 0.6	2.5 ± 2.1
Mean illness duration (days) ± SD	3.6 ± 3.8	2.9 ± 1.6	3.6 ± 3.9
Joint pain: proportion of patients reporting symptom	0.218	0.083	0.219
Peripheral Neuropathy: proportion of patients reporting symptoms	0.045	0.0	0.046

The mean body temperature of patients sampled in this study was 38.0 ± 0.5 (median = 38.0, range: 37.5–41). The mean body temperature was higher for patients belonging to ONNV positive pools than those in ONNV negative pools (Table 4). In total, 21.8% (*n* = 498) of the patients experienced joint pain, and the proportion was higher among those belonging to ONNV positive pools (Table 4). Nearly 5% of the patients complained of peripheral neuropathy symptoms, and the proportion was higher among those in ONNV positive pools (Table 4). The duration of illness and fever was variable between patients and was not different between patients infected with either ONNV or ZIKV (Table 4).

### Temporal variation in infections

**Figure 2.**
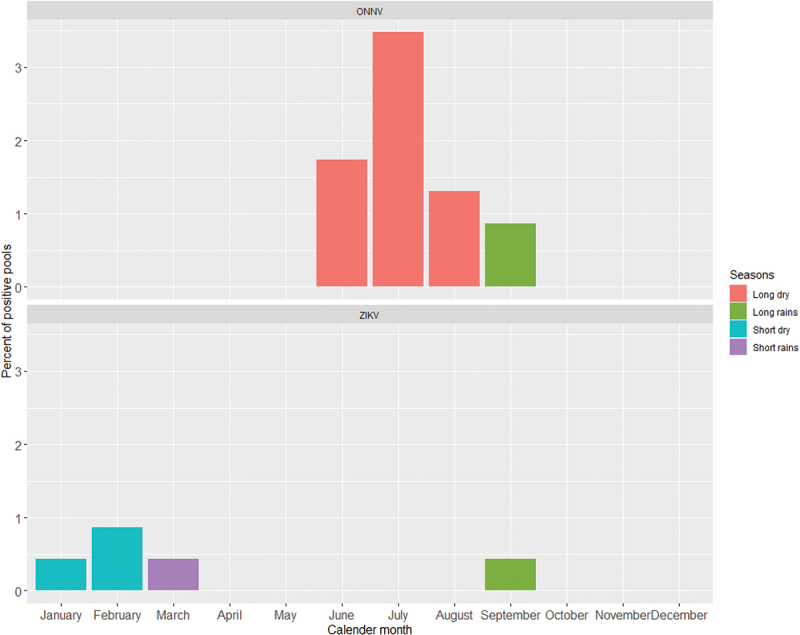
Temporal variations in ONNV (upper panel) and ZIKV (bottom panel) infections in Rwanda as a proportion of the population sampled.

ONNV and ZIKV infections varied by season, and all positive pools were sampled during 2020. In terms of seasonality, the ONNV sample pools were mainly (89.2%) from visits to the health centres during the long dry season (Figure 2, upper panel). On the other hand, the ZIKV positive pools were sampled across several seasons with infections highest during the short rains (Figure 2, bottom panel).

## Discussion

This study revealed the circulation of ONNV and ZIKV viruses among febrile patients in Rwanda during August 2019 to December 2020. There was an absence of CCHFV, CHIKV, DENV, RVFV, and WNV viruses in circulation in this period. These findings agree with previous studies from Rwanda, which detected antibodies against ONNV and CHIKV (that cross-reacts in antibody detection) in 63% of blood donors [20], and ZIKV antibodies in 1.4% [21]. However, RVFV which was previously reported in livestock in Rwanda, was not found in febrile patients [22]. Similarly, previous studies reported exposure of blood donors in Rwanda to WNV, while our study revealed absence of active WNV infection in febrile patients in Rwanda [20]. The spatial predictors of WNV is the presence of wetlands for birds and a higher density of poultry [23]. These findings suggests that the circulation of the above arboviruses during the time period was relatively low, and the occurrence is most probably influenced by climatic periodicity that drives the presence and abundance of the vectors [24].

For the viruses detected in the febrile patients, there were spatial variations. ONNV infected pools were found only in Rwaniro health centre, in the Southern province, and ZIKV cases were found in Zaza and Kirinda health centres, in East and West Rwanda, which suggested local spread in the catchment areas. In the previous antibody survey of CHIKV/ONNV in Rwanda, the seroprevalence was widespread [20], while ZIKV seroprevalence was mainly in the south-eastern region of Rwanda [21]. This variation in exposure and acute infection suggest spatio-temporal dynamics in mosquito abundance, dependent on climatic periodicity, and/or number of susceptible hosts [25,26]. For example, ONNV is transmitted by *Anopheles* mosquitoes (the malaria vector) while ZIKV is transmitted by *Aedes* mosquitoes, and these species may differ in their survival and propagation depending on habitats and climatic conditions.

The study also revealed that ONNV occurred during the dry season, in agreement with findings from Uganda, where an ONNV outbreak occurred in the dry season [27]. ZIKV cases in the study occurred in both seasons with the highest infection occurring during the short rains, and while there is strong evidence from mathematical models for periodicity in ZIKV outbreaks, it is not clearly stated whether these outbreaks occur in the wet or dry season [28]. It has been shown in some cases that vector abundance has a forcing on disease infection and abundance is linked to seasonal changes in climatic variables. For the case of ZIKV, sexual transmission among couples can dampen seasonality in transmission as secondary transmission through sex is not limited by seasons [29].

The higher prevalence in farmers may suggest occupational exposure, which is supported by a previous study on ONNV seroprevalence [30]. On the other hand, the selected health centres are in rural areas with predominance of farmers, which can explain the high participation of farmers. Interestingly, there were more cases of ONNV with high body temperature, joint pains, and peripheral neuropathy, confirming that joint pain is an important case criterion for ONNV diagnosis [31]. Although acute optic neuropathy has been associated with a severe form of CHIKV (closely related to ONNV) [32], we did not investigate optic neuropathy, during this study but our data does suggest a connection between peripheral neuropathy and ONNV. No clinical symptoms were found to be associated with ZIKV positives, although the number of ZIKV positives was too low to make any conclusions and all individuals in positive pools may have not been positive.

This study presents the circulation of selected viruses causing acute febrile illnesses in the Rwandan population. The study confirmed presence of ONNV and ZIKV in Rwanda, and could detect acute infection in patients, and for ONNV the known clinical symptoms matched with virus diagnosis. Although PCR could be used to detect, acute infections, this approach is limited if the patient low or shorting viremia. IgM serology could be used as a more sensitive alternative for detection. However, cross-reactions of antibody responses against close virus relatives is a major limitation of this method. The analysis was performed in pools, and there were no resources to analyse the individual samples. In addition, information on arbovirus notification, vector species distribution, and abundance in Rwanda is limited. However, stored samples could be analysed for different viruses and antibodies at a later stage when resources are available.

In conclusion, regular surveillance of arbovirus transmission related to vectors, reservoirs, ecology, and weather is crucial to be able to inform public health decisions for early warning, rapid response, and effective control. The finding that the Rwandan population seeks for medical care for ONNV and ZIKV infection recall for strengthening capacity for diagnostics in clinical laboratories as well as systems for surveillance, prevention, and control of emerging viral infections in Rwanda.

## Supplementary Material

Supplemental Material

## Data Availability

ONNV sequences from this study are provided in the supplementary materials to this paper.
