# Supplementary material for: Arbovirus surveillance in febrile patients attending selected health facilities in Rwanda
Source: Infect Ecol Epidemiol. 2023 Dec 7;14(1):2289872. doi: 10.1080/20008686.2023.2289872 (PMC11967279; doi:10.1080/20008686.2023.2289872)
Supplement: Supplemental Material [file ZIEE_A_2289872_SM6086.docx]

Supplementary data: Trimmed ONNV Sequences

>EGK269

TTTGCACTTGTATCGTACTGTCTGGCCGTCAACTGTGATCTTAACGTTTCCGCACCGCCCCGCCCCGCCC

>EGK271

TTTGCACTTGTATCGTACTGTCTGGCCGTCAACTGTGATCTTAACGTTTCCGCCCCGCCCCGCCCCGCCC

>EGK273

TTTGCACTTGTATCGTACTGTCTGGCCGTCAACTGTGATCTTAACGTTTCCGCCCCGCCCCGCCCCGCCC

>EGK275

TTTGCACTTGTATCGTACTGTCTGGCCGTCAACTGTGATCTTAACGTTTCCGCACCGCCCCGCCCCGCCC

>EGK292

TTTGCACTTGTATCGTACTGTCTGGCCGTCAACTGTGATCTTAACGTTTCCGCACCGCCCCGCCCCCACC

>EGK294

TTTGCACTTGTATCGTACTGTCTGGCCGTCAACTGTGATCTTAACGTTTCCGCACCGCCCCGCCCCGACC

>EGK296

TTTGCACTTGTATCGTACTGTCTGGCCGTCAACTGTGATCTTAACGTTTCCGCACCGCCCCGCCCCGCCC

>EGK410

TTTGCACTTGTATCGTACTGTCTGGCCGTCAACTGTGATCTTAACGTTTCCGCCCCGCCCCGCCCCGCCC
